# Intrinsic resistance of HIV-2 and SIV to the maturation inhibitor GSK2838232

**DOI:** 10.1371/journal.pone.0280568

**Published:** 2023-01-18

**Authors:** Robert A. Smith, Dana N. Raugi, Robert S. Nixon, Jennifer Song, Moussa Seydi, Geoffrey S. Gottlieb, on behalf of the University of Washington-Senegal HIV-2 Study Group

**Affiliations:** 1 Center for Emerging and Reemerging Infectious Diseases, University of Washington, Seattle, Washington, United States of America; 2 Department of Medicine, Division of Allergy and Infectious Diseases, University of Washington, Seattle, Washington, United States of America; 3 Service des Maladies Infectieuses et Tropicales, CHNU de Fann, Dakar, Senegal; 4 Department of Global Health, University of Washington, Seattle, Washington, United States of America; Shanxi University, CHINA

## Abstract

GSK2838232 (GSK232) is a novel maturation inhibitor that blocks the proteolytic cleavage of HIV-1 Gag at the junction of capsid and spacer peptide 1 (CA/SP1), rendering newly-formed virions non-infectious. To our knowledge, GSK232 has not been tested against HIV-2, and there are limited data regarding the susceptibility of HIV-2 to other HIV-1 maturation inhibitors. To assess the potential utility of GSK232 as an option for HIV-2 treatment, we determined the activity of the compound against a panel of HIV-1, HIV-2, and SIV isolates in culture. GSK232 was highly active against HIV-1 isolates from group M subtypes A, B, C, D, F, and group O, with IC_50_ values ranging from 0.25–0.92 nM in spreading (multi-cycle) assays and 1.5–2.8 nM in a single cycle of infection. In contrast, HIV-2 isolates from groups A, B, and CRF01_AB, and SIV isolates SIV_mac239_, SIV_mac251_, and SIV_agm.sab-2_, were highly resistant to GSK232. To determine the role of CA/SP1 in the observed phenotypes, we constructed a mutant of HIV-2_ROD9_ in which the sequence of CA/SP1 was modified to match the corresponding sequence found in HIV-1. The resulting variant was fully susceptible to GSK232 in the single-cycle assay (IC_50_ = 1.8 nM). Collectively, our data indicate that the HIV-2 and SIV isolates tested in our study are intrinsically resistant to GSK232, and that the determinants of resistance map to CA/SP1. The molecular mechanism(s) responsible for the differential susceptibility of HIV-1 and HIV-2/SIV to GSK232 require further investigation.

## Introduction

During retroviral replication, newly-generated virions undergo a series of protein processing events that convert immature particles to their mature, infectious form (reviewed in references [1–3]). In HIV-1, the structural components of the virus – matrix (MA), capsid (CA), and nucleocapsid (NC) – are primarily derived from the 55-KDa Gag precursor protein (Pr55Gag) (reviewed in references [3] and [4]). The individual folded domains and spacer peptides (SP) of Pr55Gag are linked in the following N-terminal to C-terminal order: MA-CA-SP1-NC-SP2-P6. Approximately 5% of the Gag proteins in HIV-1 are derived from a second, 160-KDa precursor (Pr160Gag-Pol). This larger precursor is produced by a ribosomal slippage event during translation of the same RNA species that encodes Pr55Gag [5]. Maturation occurs at or shortly after viral budding and is catalyzed by HIV-1 protease, which liberates the individual MA, CA, and NC proteins from Pr55Gag and Pr160Gag-Pol (reviewed in references [1, 2, 4] and [6]). Although all of the Gag cleavage events are essential for viral infectivity, the separation of CA and SP1 plays an especially pivotal role in HIV-1 morphogenesis. Cleavage of CA-SP1 appears to act as a molecular switch that triggers the rearrangement of the HIV-1 capsid lattice, resulting in the formation of mature, cone-shaped cores [7–10]. This crucial step is the target of an expanding class of antiretroviral compounds that are collectively known as maturation inhibitors (reviewed in references [6] and [11]). In this report, we use CA-SP1 to denote the immature, uncleaved form of the capsid protein, and CA/SP1 to denote the corresponding the protease cleavage site (including four amino acids on either side of the scissile peptide bond).

**Fig 1:**
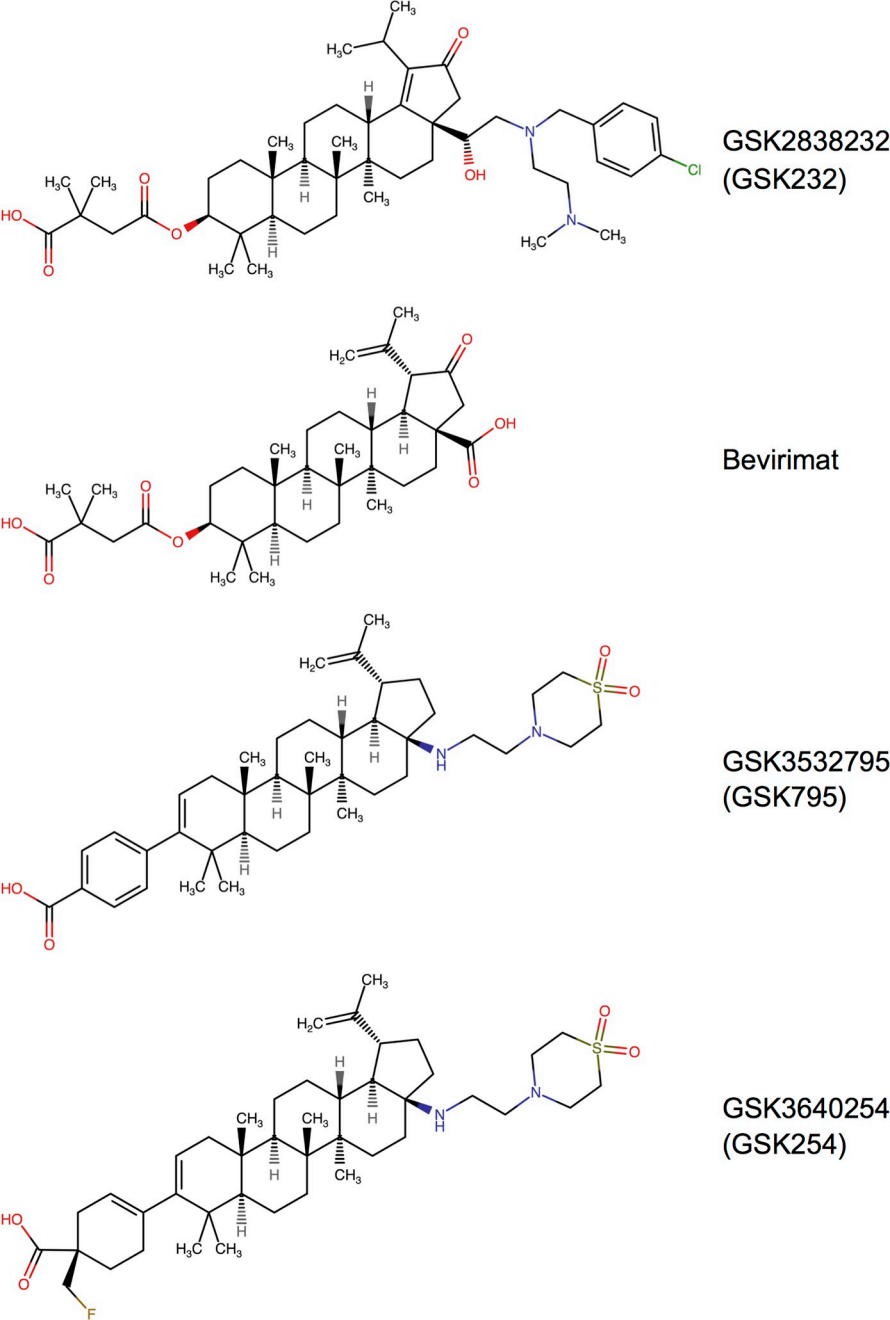
Chemical structures of the HIV-1 maturation inhibitors discussed in this study. The structure of GSK232 was adopted from Johnson et al. (70). Bevirimat was adopted from PubChem CID entry 457928. GSK795 was based on the structure reported by Nowicka-Sans et al. (49). GSK254 was adopted from Dicker at al. (45). All structures were drawn in Marvin JS version 21.14.0 (ChemAxon Int., International; available at https://chem.nlm.nih.gov/chemidplus).

Bevirimat (3-*O*-(3´,3´-dimethylsuccinyl) betulinic acid, formerly known as YK-FH312, DSB, and PA-457) (Fig 1) was the first maturation inhibitor developed for antiretroviral therapy of HIV-1 infection [12–14]. A series of biochemical and in vitro studies involving HIV-1 and SIV_mac_ helped to lay the foundation for our understanding of how bevirimat and related compounds inhibit HIV-1 replication. Analyses of virions from bevirimat-treated, HIV-1–infected cell cultures [15, 16], together with data from cell-free assays utilizing culture-derived HIV-1 cores [17] or in vitro CA assemblies [18], showed that bevirimat blocks or delays the proteolytic cleavage of CA-SP1, and that this inhibitory activity targets multimeric Gag assemblies as opposed to soluble Gag monomers. Bevirimat-resistant HIV-1 mutants selected in culture encoded amino acid changes at the P1, P1´, and P3´sites of CA/SP1 [15, 19]; these resistance-conferring changes reduce the binding of radiolabeled bevirimat to immature HIV-1 virions and virus-like particles [20, 21] suggesting that the compound might directly bind directly to CA/SP1. Additional support for a direct physical association between bevirimat and CA-SP1 was obtained in a cross-linking study using HIV-1 cores and a photoaffinity-labeled analog of the inhibitor [22]. Furthermore, SIV_mac239_ and SIV_mac251_ were shown to be highly resistant to bevirimat [15, 16, 23], but a chimeric SIV_mac239_ construct encoding the CA and SP1 regions of HIV-1_HXB2_ [24] was sensitive to the compound [16]. The determinants of bevirimat resistance in SIV_mac239_ were subsequently mapped to three amino acid residues in CA/SP1 [23], providing further evidence that the bevirimat binding site overlaps the CA/SP1 cleavage site in HIV-1 Gag.

Additional details regarding the mechanism of action of bevirimat have emerged from recent studies of the structural basis of HIV-1 maturation. In Gag assemblies that represent the immature form of HIV-1, the region spanning the C-terminal domain of CA and Sp1 exists in a dynamic equilibrium between two conformational states: a random coil, and an extended α-helix [10, 25]. Intermolecular contacts between the “junction-helix” structures of adjacent CA-SP1 monomers result in the formation of a tightly-packed, six-helix bundle (6HB) at the base of each Gag hexameric unit [10, 26–32]. Proteolytic cleavage at CA/SP1 requires the open, random-coil configuration of the peptide; in the 6HB form, the cleavage site is internalized within the bundle and is inaccessible to HIV-1 protease [8, 29, 32]. Bevirimat appears to stabilize the 6HB conformation, thereby shifting the equilibrium towards the form of CA-SP1 that is resistant to protease cleavage [31–36]. The precise orientation of bevirimat and related analogs in the 6HB is the subject of ongoing studies [22, 34, 35].

Phase I and Phase II clinical trials of bevirimat provided the first evidence that an inhibitor targeting HIV-1 maturation could produce substantial reductions in HIV-1 RNA viral load in humans [37–39]. However, during phase II studies, virologic non-responsiveness was observed in significant numbers of participants receiving the investigational compound [39, 40]. Non-responsiveness was attributed to the presence of naturally-occurring polymorphisms in SP1 that diminish bevirimat activity [20, 39, 41–44]. Subsequent drug development efforts have focused on identifying betulinic acid derivatives that are less vulnerable to polymorphisms in CA and SP1 [45–51]. GSK3532795 (GSK795; Fig 1), exhibits broader antiviral activity against polymorphic HIV-1 isolates in culture [49], but the development of GSK795 was discontinued after high rates of adverse gastrointestinal events and treatment-emergent resistance were observed in a phase IIb trial [52]. Two other bevirimat analogs, GSK2838232 (GSK232) and GSK3640254 (GSK254) (Fig 1) have been reported to have an improved activity profile relative to earlier-generation maturation inhibitors [45, 51]. GSK232 is 300-fold more potent than bevirimat in spreading (multi-cycle) infections of MT-4 cells, and retains full activity against bevirimat-resistant Gag V370A mutants of HIV-1 [51]. In phase IIa trials, administration of GSK232 plus cobicistat at doses of GSK232 ≥50 mg, or GSK254 alone at doses ≥40 mg, resulted in ≥1-log_10_ reductions in HIV-1 RNA levels; no serious safety concerns were noted for either compound [46, 53]. GSK254 is being further evaluated in two phase IIb studies (clinicaltrials.gov NCT04493216 and NCT04900038). Other novel inhibitors of HIV-1 maturation have been described, including bevirimat analogs [47, 50, 54, 55] and compounds that are structurally unrelated to bevirimat [56], but to our knowledge, these inhibitors are not currently being tested in clinical trials.

While there is an extensive body of literature regarding the activity of maturation inhibitors against HIV-1, only a few studies have examined the activity of maturation inhibitors against HIV-2. Bevirimat has been shown to have poor or no detectable activity against group A HIV-2 isolates ROD, CBL20, CBL23, and KR.X3 [15, 49, 57]. In contrast, Nowcka-Sans and colleagues reported that GSK795 was highly active against HIV-2_ROD_, with an IC_50_ value of 15 nM (versus 0.9–11 nM for seven HIV-1 isolates) [49]. In addition, in a study published earlier this year, GSK795 and GSK254 potently inhibited HIV-2_ROD_ replication in MT-2 cells when reverse transcriptase activity was used as the assay endpoint (IC_50_s, 17 and 2.5 nM, respectively) [45]. However, in this same study, both GSK795 and GSK254 were ineffective against HIV-2_ROD9_ when viral replication was measured in CEM-luciferase reporter cells (IC_50_s, >3000 nM) [45]. GSK795 also showed weak activity against HIV-2_287_ in the CEM-luciferase assay (IC_50_, 751 nM), whereas GSK254 was inactive or weakly active against HIV-2_287_ in both CEM-luciferase and MT-2 cell cultures (IC_50_s, 1245 nM and >1386 nM, respectively) [45]. Collectively, these data indicate that the activity of newer bevirimat derivatives against HIV-2 is unresolved, and warrants further study.

To assess the potential utility of a novel, HIV-1–active maturation inhibitor for the treatment of HIV-2 infection, we determined the antiviral activity of GSK232 against a panel of HIV-2 isolates using culture-based drug susceptibility assays. HIV-1 isolates from groups M and O, as well SIV_mac239_, SIV_mac251_, and SIV_agm.sab-2_ were included in our analysis as controls and comparator strains.

## Materials & methods

### Inhibitor

GSK232 was purchased from Cayman Chemical Company (Ann Arbor, MI). A master stock of 1.235 mM GSK232 was prepared by dissolving 5 mg of the compound in 500 μl of high performance liquid chromatography-grade dimethyl sulfoxide packaged under argon gas (Alfa Aesar Co., Ward Hill, Massachusetts). Serial dilutions of GSK232 ranging from 0.1 nM to 4 μM were prepared in molecular-grade water (Fisher Scientific, Fair Lawn, NJ), yielding working stocks with GSK232 concentrations 10-fold greater than the final concentrations used in culture. All stocks of GSK232 were stored at –80°C and were thawed at 37°C immediately before use.

### Cell lines & media

CEMss cells and MOLT-4 clone 8 cells were obtained from the National Institutes of Health (NIH) HIV Reagent Program, Division of AIDS, National Institute of Allergy and Infectious Diseases. 293tsA1609neo clone 17 cells (293T/17) were obtained from the American Type Culture Collection (Manassas, VA). HeLa-CD4-CCR5-LTR-β-galactosidase indicator cells (MAGIC-5A) were a kind gift from Michael Emerman, Fred Hutchinson Cancer Research Center (Seattle, WA). CEMss cells were grown in Iscove’s modified Dulbecco’s medium (IMDM) with GlutaMAX™-I (Gibco, Life Technologies Corp., Grand Island, NY; catalog number 31980-030). MAGIC-5A and 293T/17 cells were grown in Dulbecco’s modified Eagle’s medium (DMEM) (Corning-Mediatech, Manassas, VA; catalog number 10-017-CM). Both media were supplemented with 4 mM L-glutamine, 100 U/ml penicillin, 100 μg/ml streptomycin (Gibco, Life Technologies Corp., Grand Island, NY), and fetal bovine serum (MilliporeSigma, St. Louis, MO). The final concentration of serum in complete medium was 10% vol/vol for DMEM, and 15% vol/vol in IMDM. Fetal bovine serum was heat-inactivated (70°C for 30 min) and stored in 25-ml aliquots at −20°C.

### HIV-1, HIV-2, and SIV isolates and molecular clones

HIV-1_92UG029_, HIV-1_93BR020_, HIV-1_BCF01_, HIV-2_CDC310319_, and SIV_agm.sab-2_ were obtained from the NIH HIV Reagent Program as cell-free virus stocks. SIV_agm.sab-2_ was cultured in MOLT-4, clone 8 cells; isolates 92UG029, 93BR020, BCF01, and CDC310319 were cultured in CEMss cells to obtain stocks for drug susceptibility testing. The remaining virus isolates tested in this study were generated via transient transfection of full-length, plasmid molecular clones into 293T/17 cells, as described below. Plasmid pROD9 was provided by Michael Emerman. The pNL4-3 molecular clone was obtained from Bruce Chesebro, National Institutes of Health (NIH), Rocky Mountain Laboratories, Hamilton, MT. Plasmid p7312A-JK was a kind gift from George Shaw, University of Pennsylvania, Philadelphia, PA. Plasmids pQ23-17, pMJ4, p94UG114.1.6, pHIV-2/ST, pSpX (encoding SIV_mac239_) and pBK28 (encoding SIV_mac251_) were obtained from the NIH HIV Reagent Program. Plasmids were maintained in Escherichia coli strains XL10-Gold (Agilent Technologies; Santa Clara, CA) or TOP-10 (Invitrogen Life Technologies; Carlsbad, CA). All bacterial growth was performed at 30°C to minimize the outgrowth of plasmids with deletions in the virus-encoding sequences [58]. Transfection-grade preparations of each plasmid were produced using a HiSpeed^®^ Plasmid Maxi Kit, with buffers and reagents from an EndoFree^®^ Plasmid Maxi Kit (Qiagen Co.; Valencia, CA). DNA concentrations were determined using a NanoDrop™ ND-1000 Spectrophotometer (Thermo Fisher Scientific; Waltham, MA).

### Transfections for generating virus stocks

293T/17 cells were seeded into 6-well plates at a density of 5 × 10^5^ cells per well in 2 ml of complete DMEM and placed in a humidified incubator at 37°C in 5% CO_2_ for approximately 22 hours. Thirty minutes prior to transfection, each well was treated with chloroquine by adding 4.2 μl of a 10 mM solution of chloroquine diphosphate salt (Sigma-Aldrich Co., St. Louis, MO) that was prepared beforehand in molecular biology-grade water (Fisher Scientific; Fair Lawn, NJ). Calcium phosphate-DNA co-precipitates were prepared in 1.8-ml micro-centrifuge tubes by adding 6 μg of plasmid DNA to 300 μl of 0.2 M CaCl_2_, and then adding this mixture drop-wise to 300 μl of HEPES-buffered saline solution (270 mM NaCl, 10 mM KCl, 1.5 mM Na_2_HPO_4_ · 2H_2_O, 11 mM dextrose, 40 mM HEPES, pH 7.05). The tube containing HEPES buffer was agitated with a Standard Vortex Mixer (Fisher Scientific, speed setting: 6) during the addition of DNA in CaCl_2_. The final co-precipitate products were added to three wells of chloroquine-treated 293T/17 cells (200 μl per well), and the culture plates were returned to the incubator. Approximately 18 hours after the addition of DNA, the supernatant in each well was removed and replaced with 2 ml of fresh, complete DMEM. Growth was then continued overnight, and culture supernatants were collected on the second day after DNA addition. All supernatants were clarified by centrifugation in a Beckman GPR centrifuge with a GH37 swinging-bucket rotor for 10 min at 1,000 rpm at 22°C. Clarified supernatants were stored at −80°C.

### Virus titration in MAGIC-5A cells

Sub-confluent monolayers of MAGIC-5A cells were rinsed with Dulbecco’s phosphate-buffered saline solution without calcium or magnesium (D-PBS; HyClone Laboratories, Inc., Logan, UT), disrupted with 0.05% trypsin–EDTA (Gibco), and seeded into 48-well tissue culture plates as previously described [59]. Each well received 1.5 × 10^4^ cells in 200 μl of complete DMEM. The outer wells of the plates received 300 μl of D-PBS to avoid “edge effects” on cell growth and viability [60]. MAGIC-5A assay plates were incubated for 18 to 20 h at 37°C in 5% CO_2_ prior to infection. To titrate the virus stocks, serial 4-fold dilutions of each strain were prepared in complete DMEM containing 20 μg/ml diethylaminoethyl-dextran (DEAE-dextran; Sigma-Aldrich Co., St. Louis, MO). The medium was then aspirated from the assay plates, and the diluted virus preparations were added directly to the MAGIC-5A cell monolayers (100 μl/well). After a 2-h incubation at 37°C in 5% CO_2_, 300 μl of complete DMEM was added to each well, and the plates were returned to the incubator for 40 to 44 h. Infected monolayers were treated with fixative, washed twice D-PBS, and stained with 5-bromo-4-chloro-3-indolyl- β-D-galactopyranoside (X-gal) as previously described [61]; β-galactosidase positive foci were counted by light microscopy. Titers (presented as the number of focus-forming units [FFU] per milliliter) were back-calculated on the basis of the dilution of virus that yielded 50 to 200 foci per well.

### Virus titration in CEMss cells

All virus isolates that were evaluated for GSK232 susceptibility in the spreading infection assay (see below) were first titrated in CEMss cells using a limiting dilution approach. CEMss cells from continuously-replicating cultures were seeded in 96-well Nunc™ Edge 2.0 microtiter plates (Thermo Fisher Scientific) at 2 × 10^5^ cells per well in 150 μl per well of complete IMDM containing 20 μg/ml DEAE-dextran. Each of the four outer reservoirs of the Edge 2.0 plates was filled with 1.5 ml of sterile D-PBS. Serial ten-fold dilutions of each virus stock were prepared in complete IMDM, and 50 μl per well of the dilutions were added to the CEMss plates. Four culture wells were infected for each dilution of virus; the dilutions ranged from 1:10 to 1:10^12^. After the addition of virus, the assay plates were placed at 37°C in 5% CO_2_ for 12 days. On the second, fourth, sixth, eighth, and tenth days of incubation, the micro-cultures were mixed by pipetting, 110 μl of culture fluids were removed, 110 μl of fresh complete IMDM were added to each well, and the plates were returned to the incubator. All CEMss plates were stored at −80°C at the end of the incubation period. To score the wells as positive or negative for infection, we thawed the plates at 37°C, and transferred samples of the cultures (50 μl per well) to MAGIC-5A cells that were seeded in 96-well Edge 2.0 plates. The MAGIC-5A plates were then stained with X-gal after 44 h of growth as previously described. For these assays, the MAGIC-5A cells were seeded one day prior to infection at 5 × 10^3^ cells per well in 200 μl per well of culture medium. Titers of the original virus stocks used to infect the CEMss cells were calculated as the 50% tissue culture infectious dose (TCID_50_) using the method of Reed and Muench [62].

### Site-directed mutagenesis

CA/SP1 mutant HIV-2_ROD9_ M3 was constructed using the pROD9 molecular clone and reagents from a QuikChange II XL Site-directed Mutagenesis Kit (Agilent Technologies). The mutant clone was constructed in two stages. First, Gag amino acids leucine 363 and methionine 364 (numbered according to the Los Alamos HIV Database HIV-2/SIV_sm_ consensus alignment; [63]) were changed to valine and leucine, respectively, using oligonucleotides 5´-CAGAAAGCTAGAGTATTGGCAGAGGCCCTG-3´and 5´-CAGGGCCTCTGCCAATACTCTAGCTTTCTG-3´ (mutant nucleotides underlined). Next, a leucine to methionine change at position 368 of Gag was introduced into the clone produced by the first round of mutagenesis reactions using oligonucleotides 5´-**T**TGGCAGAGGCCATGAAAGAGGTCATAGG-3´ and 5´- CCTATGACCTCTTTCATGGCCTCTGCCA**A**-3´. This second pair of oligonucleotides preserved the point mutation that encoded the methione 364 to leucine change, which was introduced into pROD9 in the first round of reactions (nucleotide bases indicated in bold type). The nucleotide sequence of the resultant clone, HIV-2_ROD9_ M3, was confirmed via automated Sanger DNA sequencing (Azenta Life Sciences Inc., Seattle, WA) using primer ROD#35 (nucleotide sequence, 5´-CCAAAGCTATGTAGATAGATTCTA-3´).

### Single-cycle drug susceptibility assays

Single-cycle measurements of GSK232 susceptibility were performed using a protocol that is similar to our method for determining HIV-1 protease inhibitor sensitivity [64], with a few modifications. This approach involves two culture steps: virus production in transiently-transfected, GSK232-treated 293T/17 cells, and quantification of the levels of infectious virus released into the 293T/17 supernatants using MAGIC-5A indicator cells. In the first phase of the assay, 293T/17 cells were seeded into 48-well, tissue culture-treated plates (two plates per virus isolate) at a density of 3 × 10^4^ cells per well in 200 μl of complete DMEM per well, and incubated for ~24 h at 37°C in 5% CO_2_. Immediately prior to transfection, the culture wells were aspirated, and the medium was replaced with 200 μl per well of fresh, complete DMEM. The cultures were then treated with 25.5 μl per well of appropriate dilutions of GSK232 (or molecular-grade water for solvent-only and no-DNA controls) and 10 μl per well of 420 μM chloroquine diphosphate salt (for all culture wells) and returned to the incubator. Two batches of calcium phosphate-DNA co-precipitates were prepared for each virus clone as described above; the co-precipitates were pooled and added to the chloroquine-treated 293T/17 cells (20 μl per well), and the plates were incubated for an additional 44 h. After this time, the upper 110 μl of supernatant was collected from each assay well, transferred to non-tissue culture treated, 96-well plates and stored at −80°C. In the second phase of the assay, samples of the 293T/17 supernatants (50 μl per well) were transferred to MAGIC-5A cultures to quantify infectious virus. Conditions for the seeding and infection of MAGIC-5A cells were identical to those described above for the MAGIC-5A virus titrations, except that the plates were aspirated and replenished with 250 μl per well of complete DMEM containing 20 μg/ml DEAE-dextran prior to the addition of virus (as opposed to adding the virus-containing samples directly to the monolayers). Because infectious virus production and virus-induced syncytia formation can vary markedly between different HIV and SIV molecular clones, it was necessary in some cases to dilute the 293T/17 supernatants prior to their use in infecting the MAGIC-5A cells to avoid syncytial lysis. In such cases, the 293T/17 supernatants were diluted in complete DMEM at a constant, fixed ratio across the entire dose-response series (including samples from the no-drug control wells); this was done immediately prior to the addition of the 293T/17 supernatants to the MAGIC-5A assay plates. Furthermore, after 40-h of incubation, the MAGIC-5A plates were inspected via light microscopy to ensure that the level of virus-induced syncytia in the no-drug wells was ≤25% of the total cells in monolayer; plates with >25% syncytia were discarded and were not analyzed further. Following the 40-h incubation period, the MAGIC-5A plates were lysed and treated with chlorophenol red-β-D-galactopyranoside (CPRG) to quantify β-galactosidase activity as previously described [59]. Absorbance was measured at 570 nm using a Victor^3^ Multilabel Plate Reader (PerkinElmer Inc., Akron, OH). Absorbance readings for the HIV/SIV-infected culture wells were background-corrected by subtracting the mean absorbance obtained in mock-transfected (no-DNA) control wells. Background-corrected readings were then used to calculate “% of no-drug control” values by dividing the absorbance in each drug-treated well by the mean absorbance observed in the no-drug control wells, and multiplying the quotient by 100. This normalization step is conventional in antiviral dose-response experiments and does not affect the interpretation of data for highly resistant isolates or strains (i.e., the absence of an inhibitory effect is apparent in both the raw data and in the normalized absorbance measurements). The resultant “% of no-drug control” values were plotted as a function of the log_10_ drug concentration, and 50% inhibitory concentrations (IC_50_ values) were calculated using a four-parameter regression model in Prism version 6.0h (GraphPad Software, Inc., San Diego, CA).

### Spreading-infection drug susceptibility assays

Drug sensitivity measurements in CEMss cells were performed using a modified version of our previously-described protocol [65]. CEMss cells were seeded into EDGE 2.0 96-well plates at 2.3 × 10^5^ cells per well in 150 μl per well of complete IMDM containing 20 μg/ml DEAE-dextran. GSK232 or water for the no-drug controls was added (22 μl per well, 4 wells per drug concentration), and the plates were incubated at 37°C in 5% CO_2_ for 60 min. Infections were initiated with 50 μl per well of cell-free virus; four wells of the assay plate received medium only (no-virus controls). The cells were diluted and supplemented with fresh medium on days two and four after infection as described above for the CEMss titration assay, except that each well received 110 μl of medium and 11 μl of appropriate GSK232 stock (or water for the no-virus and no-drug controls). These re-feeding and re-dosing steps were performed in order to maximize the viability of the CEMss cells throughout entire culture period. In addition, the GSK232 stocks that were used to re-dose the plates on days 2 and 4 were the same as those used to dose the plates on day 0. In this way, the final concentration of GSK232 in the culture wells was maintained as consistently as possible throughout the duration of the assay. On the sixth day of infection, the plates were frozen at −80°C. To quantify infectious virus production, the CEMss assay plates were thawed for approximately 30 min at 37°C, 5% CO_2_, and samples of the CEMss cultures (30 μl per well) were transferred to MAGIC-5A cells that had been seeded one day prior in 96-well plates as described above for the CEMss titration assay. The MAGIC-5A cells were then lysed and treated with CPRG as previously described [59], and absorbance at 570 nm was measured using the Victor^3^ plate reader. The resultant readings were background-corrected, normalized, and plotted as described for the single-cycle drug susceptibility assay (see above) to determine IC_50_ values for each virus isolate.

### Cytotoxicity assays

The toxicity of GSK232 in CEMss, 293T/17, and MAGIC-5A cells was measured using reagents from the CellTiter-Glo^®^ Luminescent Cell Viability Assay kit (Promega Corp., Madison, WI). Each cell type was seeded into 48-well or 96-well assay plates, treated with varying concentrations of GSK232 (or water for solvent-only control wells), and cultured as described above for the spreading infection (CEMss) and single-cycle (293T/17 and MAGIC-5A) drug susceptibility assays. Immediately prior to performing the viability assays, lyophilized CellTiter-Glo^®^ Substrate was reconstituted in CellTiter-Glo^®^ Buffer (to produce CellTiter-Glo Solution), and the assay plates containing cells in culture medium were equilibrated at room temperature (~30 min incubation). For the 293T/17 cells, the upper 200 μl of medium was removed from each well and discarded, and 100 μl of CellTiter-Glo Solution was added to each well. For the CEMss cells, the cultures were mixed by pipetting, and 50-μl samples (cells and medium) from each well were transferred to a new 96-well plate; 50 μl of CellTiter-Glo Solution was then added to each sample. For the MAGIC-5A cells, the entire volume of culture medium (300 μl/well) was removed, the monolayers were washed twice with 200 μl/well of room-temperature D-PBS, and 100 μl of CellTiter-Glo Solution was added directly to the washed monolayers. For all three cell lines, after adding CellTiter-Glo Solution, the plates were rocked for 2 min and incubated for an additional 10 min at room temperature, and the resultant lysates (60 μl per well) were transferred to 96-well, LUMITRAC™ assay plates (Greiner Bio-One, Monroe, NC). Luminescence was measured using the Victor^3^ plate reader. Readings from CellTiter-Glo Solution-only control wells were subtracted from the readings for each of the wells containing cell lysates, and the reading for each GSK232-treated well was normalized to the average reading from two solvent-only (no-drug) control wells from the cell culture plates. The resultant data were plotted in Prism to determine 50% cytotoxic concentrations (CC_50_s) for each of the cell lines.

### CA/SP1 sequences

We compiled the amino acid sequences of CA/SP1 for each of the HIV-1, HIV-2, and SIV isolates and clones tested in our study. The region spanning the five amino acids on either side of the CA/SP1 cleavage site corresponds to amino acids 360–369 of HIV-2 Gag, as numbered in the Los Alamos HIV Database HIV-2/SIV_sm_ consensus alignment; [63]) Sequences for clones pQ23-17, pMJ4, p94UG114.1.6, pHIV-2/ST, and pSpX were obtained from text files supplied by the NIH HIV Reagent Program (available at https://www.aidsreagent.org), and were found to be identical to sequences in GenBank for the corresponding virus isolates or molecular clones in the region of interest (GenBank accession numbers AF004885, AF321523, U88824, M31113, and M33262, respectively). The sequence for p7312A was provided by the laboratory of George Shaw via Fred Bibollet-Ruche University of Pennsylvania, Philadelphia, PA. The CA/SP1 sequence of pROD9 was determined experimentally using primer ROD#35 (primer sequence provided above). Sequences for virus isolates HIV-1_92UG029_, HIV-1_93BR020_, and SIV_agm.sab-2_, and molecular clones pNL4-3 and pBK28, were obtained from GenBank (accession numbers AY13407, AF005494, U04005, AF324493 and M19499, respectively). Sequence information was not available for HIV-1_BCF01_ and HIV-2_CDC310319_.

## Results

We initially measured the activity of GSK232 against HIV-1, HIV-2, and SIV isolates in spreading infections of an immortalized, human T cell line (CEMss). Briefly, CEMss cells were seeded into microtiter plates, dosed with varying concentrations of GSK232, infected, and cultured for six days. At the end of the six-day period, samples from the CEMss cultures were transferred to MAGIC-5A indicator cells to score infectious titers. Inhibition of viral replication was determined by quantifying β-galactosidase expression in lysates of the MAGIC-5A monolayers; this was achieved using a colorimetric substrate (CPRG) in conjunction with a semi-automated plate reader (see Materials and Methods for additional details).

Before beginning the spreading infection assays, we determined the appropriate multiplicity of infection (MOI) to use for each of the HIV and SIV isolates in our panel. Our objective was to identify a narrow range of MOIs that produced detectable titers for all isolates after six days of culture, while simultaneously avoiding excessive levels of syncytia formation and virus-induced cytopathic effects (CPE) in the CEMss cells. This precaution was taken to avoid saturation of the spreading infection assay, which might falsely inflate the IC_50_ for GSK232. In these experiments, each isolate was cultured in CEMss cells under conditions identical to those used in the drug susceptibility assays, except that the infections were initiated with serial dilutions of the virus stocks (range, 10- to 10^12^-fold in 10-fold increments. After six days of growth, we recorded the degree of syncytium formation in the CEMss cells using a qualitative score, and transferred samples from the CEMss plates to a second set of plates that were seeded with MAGIC-5A cells. These steps mirror the virus production and scoring phases of the drug susceptibility assay described below. We found that an MOI of 9.0 × 10^-3^ TCID_50_ per cell produced MAGIC-5A–detectable titers for nearly all of the viruses that were tested in the spreading infection assay (Table 1), while at the same time avoiding excessive CPE in the CEMss cultures (i.e., ≤40% of cells involved in syncytia). The lower MOIs used for HIV-1_94UG114.1.6_ and HIV-2_7312A_ (Table 1) reflect the lower titers of infectious virus in these particular virus stocks, and the maximum volume of supernatant that could be used to infect the CEMss cultures (50 μl).

**Table 1:** Susceptibility of HIV-1, HIV-2, and SIV isolates to GSK2838232 in spreading infections of CEMss cells.

Virus	Isolate	Group/ subtype	MOI (× 10^-3^)^a^	IC_50_ (nM)^b^			No. of assays
HIV-1	92UG029	M/A	9.0	0.35	±	0.013	2
	NL4-3	M/B	9.0	0.34	±	0.0088	2
	94UG114.1.6	M/D	0.82^c^	0.92	±	0.30	3
	93BR020	M/F	9.0	0.25	±	0.021	3
	BCF01	O	9.0^d^	0.79	±	0.23	3
HIV-2	ROD9	A	9.0^d^	not calculable			3
	ST	A	9.0	not calculable			3
	CDC310319	B	9.0	not calculable			3
	7312A	CRF01_AB^e^	4.0^c^	not calculable			3
SIV_mac_	239		9.0^d^	not calculable			2
	251		9.0	not calculable			3
SIV_agm_	sab-2		9.0	not calculable			3

^a^MOI, multiplicity of infection, expressed as the 50% tissue culture infectious dose (TCID_50_) per CEMss cell. TCID_50_ values were determined via endpoint dilution in CEMss cells, and were calculated via the method of Reed and Muench.

^b^IC_50_ values for the HIV-1 isolates were calculated using a 4-factor regression model in GraphPad Prism version 6.0h. IC_50_ values for HIV-2 isolates ST, CDC77618, and 7312A, as well as SIV_mac239_, SIV_mac251_ and SIV_agm.sab-2_, could not be calculated because the level of infectious virus in culture wells that were dosed with the highest concentration of GSK232 used in the assay (40 nM) did not drop below 50% (relative to untreated controls). Similar results were obtained in two of three assays with HIV-2_ROD9_; in the third experiment, the level of infectious virus at 40 nM GSK232 was 46% of that found in the no-drug control wells.

^c^Values were the maximal MOI achievable with the HIV-1_94UG114.1.6_ and HIV-2_7312A_ virus stocks.

^d^Slight adjustments to the MOIs for HIV-1_BCF01_, HIV-2_ROD9_, and SIV_mac239_ were made during the course of the experiments. For all three isolates, the final MOI differed from the one listed above by a factor of 3.0 or less.

^e^Inter-group A/B recombinant. The gag gene of HIV-2_7312A_ is monophyletic with gag sequences from group B HIV-2 isolates [66].

We also determined the toxicity of GSK232 in CEMss and MAGIC-5A cells in order to preclude the involvement of inhibitor-induced cytotoxic effects in any observed antiviral responses. As in the experiments described above, cytotoxicity was determined under conditions that were identical to those used to measure viral susceptibility to GSK232, except that an equivalent volume of culture medium was used in place of the viral inoculum. In both CEMss cells and MAGIC-5A indicator cells, viability (as measured using the CellTiter Glo assay) was >90% of the no-drug controls at GSK232 concentrations as high as 100 nM, whereas partial inhibition of CEMss and MAGIC-5A cell growth was observed at 400 nM GSK232 (S1 Fig). Based on these results, we used a maximal concentration of 40 nM GSK232 in drug-treated CEMss cultures.

**Fig 2:**
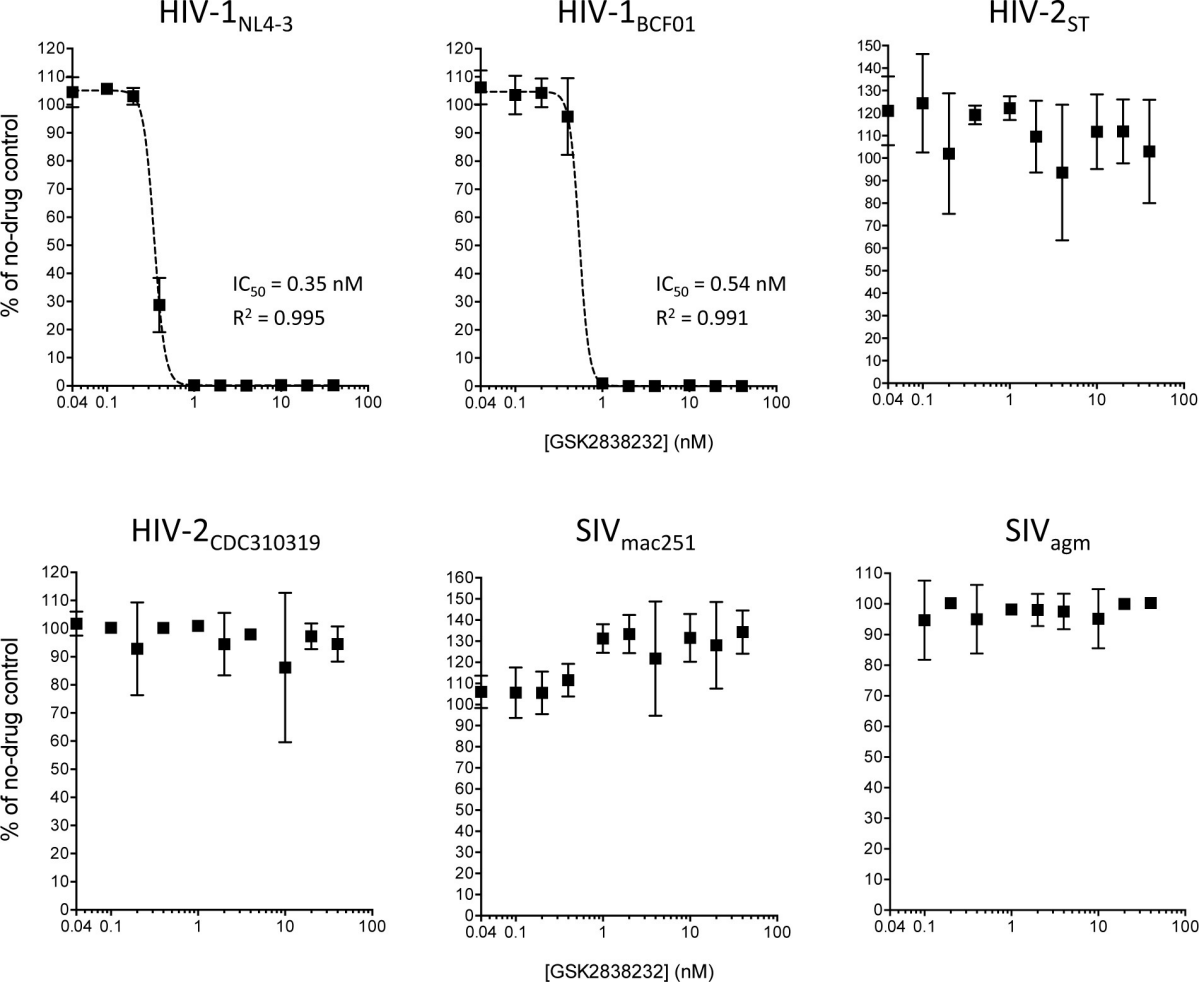
Dose-response plots from spreading-infection assays of GSK232 antiviral activity, which were performed in CEMss cells. Data points indicate the amount of infectious virus produced in GSK232-treated cultures relative to the amount produced in cultures that received solvent only (no-drug controls). Each point is the mean of four cultures that were maintained in parallel. Error bars indicate ±1 SD and, when not visible, are smaller than the symbols. IC_50_ values were calculated for the HIV-1 isolates using a four-parameter regression model in GraphPad Prism 6.0 as described in the Materials and Methods; the regression curves are shown as dashed lines. R^2^ values are also shown as calculated in Prism.

In the spreading infection assay, dose-response curves for HIV-1 isolates 92UG029, NL4-3, 92UG114.1.6, and 93BR020, (representing group M subtypes A, B, D, and F, respectively) showed a downward slope of inhibition between GSK232 concentrations of 0.2 nM and 1 nM (Fig 2 and S2 Fig). Similar results were observed for the group O HIV-1 isolate BCF01 (Fig 2). Average IC_50_ values from two or more assay runs ranged from 0.25 to 0.79 nM for the five HIV-1 isolates tested in the CEMss spreading infection assay (Table 1). In contrast, HIV-2 isolates ST (group A), CDC310319 (group B), and 7312A (CRF01_AB), and SIV isolates mac239, mac251, and agm showed no detectable sensitivity to GSK232 in CEMss cells (Table 1, Fig 2, and S2 Fig). For these HIV-2 and SIV isolates, the level of infection at the highest concentration of GSK232 used in the assay (40 nM) was >50% of that seen in no-drug control wells from the same assay plates. Similar results were observed in two of three assay runs with HIV-2_ROD9_ (group A); in the third experiment with HIV-2_ROD9_, the level of infectious virus at 40 nM GSK232 was 46% of the no-drug controls (S2 Fig).

To further evaluate the susceptibility of HIV-2 and SIV to GSK232, we performed additional experiments that quantified antiviral activity in a single round of viral replication. 293T/17 cells were treated with GSK232 and transfected with plasmids that encoded full-length HIV-1, HIV-2, and SIV genomes. The resulting yield of infectious virus was determined by plating supernatants from the 293T/17 cultures onto MAGIC-5A indicator cells. For assays involving HIV-2 and SIV, the highest concentration of GSK232 used in the 293T/17 cultures was increased to 100 nM (as opposed to 40 nM in CEMss cells), since preliminary experiments showed that the IC_50_ for GSK232, when tested against HIV-1_NL4-3_, was approximately 5–10-fold higher in the single-cycle assay in comparison to the spreading infections. This concentration of GSK232 (100 nM) was minimally toxic to both 293T/17 and MAGIC-5A cells, as determined using the CellTiter Glo assay (<20% reduction in viability relative to solvent-only controls) (S1 Fig.).

In keeping with the results observed in spreading infections of CEMss cells, GSK232 was highly active against HIV-1 isolates Q23-17, NL4-3, MJ4, and 94UG114.1.6 in the single cycle assay (S3 Fig). Mean IC_50_ values for these four HIV-1 strains ranged from 1.5 to 2.8 nM (Table 2). For HIV-2 isolates ROD9, ST, and 7312A, as well as SIV_mac239_ and SIV_mac251_, the corresponding dose-response plots tended to show a gradual decrease in the level of infectious virus at GSK232 concentrations >10 nM, suggesting that the drug exerts weak antiviral activity against HIV-2 and SIV in 293T/17 cells (S4 Fig). In two of the six assays performed with HIV-2_ROD9_, the level of infectious virus that was produced in the presence of 100 nM GSK232 was 45–47% of the level seen in the no-drug control wells (S4 Fig). For the remaining HIV-2 and SIV isolates, the level of infectious virus at 100 nM GSK232 was >50% of that seen in the no-drug controls (S4 Fig).

**Table 2:** Susceptibility of HIV-1, HIV-2, and SIV isolates to GSK2838232 in a single cycle of viral replication.

Virus	Isolate	Group/subtype	IC_50_ (nM)^a^			No. of assays
HIV-1	Q23-17	M/A	2.8	±	0.61	4
	NL4-3	M/B	2.8	±	1.1	4
	MJ4	M/C	1.5	±	0.39	3
	94UG114.1.6	M/D	2.7	±	0.35	3
HIV-2	ROD9	A	not calculable^b^			6
	ST	A	not calculable			3
	7312A	CRF01_AB^b^	not calculable			3
SIV_mac_	239		not calculable			3
	251		not calculable			4

^a^IC_50_ values for the HIV-1 isolates were calculated using a 4-factor regression model in GraphPad Prism version 6.0h. IC_50_ values for HIV-2_ST_, HIV-2_7312A_, SIV_mac239_ and SIV_mac251_ could not be calculated because the absorbance readings for these isolates (expressed as a percentage of the average absorbance obtained in no-drug control cultures) did not drop below the 50% level at the highest concentration of GSK232 used in the assay (100 nM). A similar outcome was observed in 4 of 6 experiments with HIV-2_ROD9_; in the other two assays, the absorbance readings at 100 nM GSK232 were 45–47% of the no-drug controls.

^b^Inter-group A/B recombinant. The *gag* gene of HIV-2_7312A_ is monophyletic with *gag* sequences from group B HIV-2 isolates [66].

**Fig 3:**
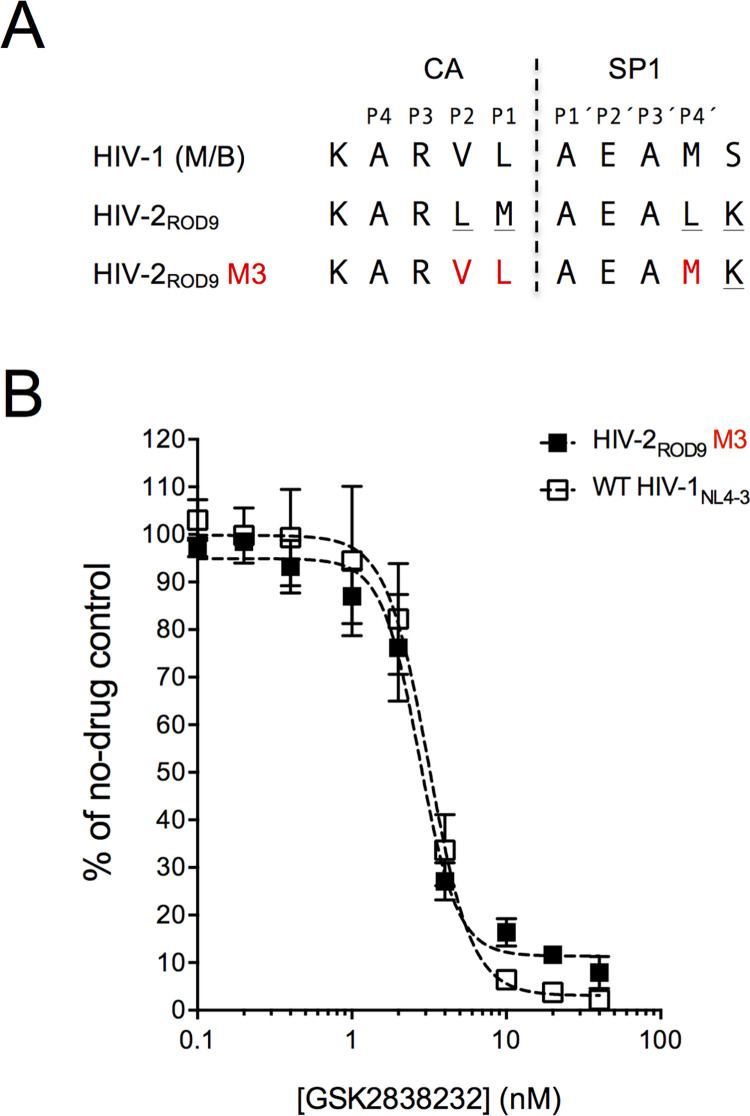
Effects of amino acid changes in the CA/SP1 site of HIV-2_ROD9_ on GSK232 susceptibility. **A** Alignment of HIV-1 and HIV-2 Gag CA/SP1 amino acid sequences. The top line (HIV-1 M/B) shows the consensus sequence from an analysis of 1295 HIV-1 group M subtype B isolates. This sequence is available at https://www.hiv.lanl.gov/content/sequence/NEWALIGN/align.html, using the following settings – Alignment type: consensus/ancestral, Organism: HIV-1/SIV_cpz_, Region: GAG, Subtype: M group without recombinants, Year: 2021. The corresponding sequence encoded by the pROD9 molecular clone of HIV-2, and a variant of pROD9 (plasmid clone M3) in which the bases encoding the P2, P1, and P4´ sites of CA/SP1 were altered via site-directed mutagenesis is also shown. All of the group M HIV-1 isolates (including molecular clones) that were tested in this study encode CA/SP1 sequences that are identical to the one shown for HIV-1 M/B except for HIV-1_92UG029_, which contains an isoleucine at the P2 position (see Materials & methods for sequence sources). HIV-2_ST_, HIV-2_7312A_, SIV_mac239_, SIV_mac251_, are identical to that of HIV-2_ROD9_ in the region shown; SIV_agm.sab-2_ contains a threonine at the c-terminal–most site in the alignment. Sequence information was unavailable for HIV-1_BFC01_ and HIV-2_CDC310319_ in the region of interest. **B** Dose-response curves from single-cycle assays with HIV-2_ROD9_ mutant M3 and HIV-1_NL4-3_. Each point is the mean of four cultures that were maintained in parallel. Error bars indicate ±1 SD and, when not visible, are smaller than the symbols. IC_50_ values were calculated for the HIV-1 isolates using a four-parameter regression model in GraphPad Prism 6.0 as described in the Materials & methods; the regression curves are shown as dashed lines. R^2^ values were also calculated in Prism. IC_50_ values for the data plotted in this figure are 2.7 nM for HIV-2_ROD9_ M3 (R^2^, 0.98) and 3.2 nM for HIV-1_NL4-3_ (R^2^, 0.97).

In a previous study, Zhou and colleagues found that three amino acid changes in the CA/SP1 site of SIV_mac239_ were sufficient to convert the virus to a form that was susceptible to bevirimat [23]. The reported IC_50_ value for the triply-substituted variant in spreading infections of human CD4^+^ T cells was ~12 ng/ml; based on a molecular weight for bevirimat of 584.8, this corresponds to an IC_50_ of ~21 nM. To determine whether the CA/SP1 sequence of HIV-2 plays a similar role in dictating GSK232 susceptibility, we constructed a mutant of HIV-2_ROD9_ in which the P2, P1, and P4´ sites of CA/SP1 were changed to the corresponding amino acids found in HIV-1 (mutant M3; Fig 3A). Titers produced by transfecting the M3 clone into 293T/17 cells (as measured by plating 293T/17 supernatants onto MAGIC-5A cells) were 25% of those produced by the parental HIV-2_ROD9_ plasmid, indicating a substantial fitness cost. However, the amount of infectious virus produced by the mutant clone was sufficient for drug susceptibility testing in the single-cycle assay. The resultant dose-response profile for M3 was similar to the profiles obtained with HIV-1 (Fig 3B). In three independent assay runs, the IC_50_ value for the M3 mutant was 1.8 ± 0.90 nM (mean ±1 standard deviation). No statistically-significant differences were detected in a comparison of the IC_50_s for HIV-2_ROD9_ M3 with the corresponding values for each of the four HIV-1 isolates tested in the single-cycle assay (*P* >0.05, analysis of variance with Tukey’s multiple comparisons test).

## Discussion

Our data for the HIV-1 isolates tested in the spreading infection assay (Table 1) are consistent with those of Jeffrey et al., who reported IC_50_ values ≤5 nM for 97 of 101 HIV-1 isolates tested in GSK232-treated peripheral blood mononuclear cells [51]. In addition, our IC_50_ values for HIV-1_NL4-3_ and other HIV-1 isolates evaluated in the single-cycle assay (Table 2) are on par with those reported for two other “second-generation” maturation inhibitors: GSK254 (IC_50_, 3 nM) and GSK795 (IC_50_, 5 nM); these data were obtained using an HIV-1_NL4-3_–based vector and a similar, single-cycle assay method [45]. We note that the IC_50_ values in our single-cycle assays with HIV-1 were, on average, approximately 5-fold higher than those seen in the spreading infections (Tables 1 and 2). A similar tendency has been seen in previous studies of HIV-1 integrase inhibitors [65, 67–69] and nucleoside reverse transcriptase inhibitors [59, 70]. As with other culture-based methods, the IC_50_ values obtained in our experiments are dependent on the specific assay conditions and cell types used to measure inhibitor susceptibility, and may not precisely reflect the inhibitory potential of GSK232 in various physiological compartments *in vivo*. However, for both of the assay methods used in our study, the conditions used to dose, infect, and culture the cells were consistent across the entire range of isolates tested, thereby providing an unambiguous comparison of HIV-1, HIV-2 and SIV isolates with respect to GSK232-mediated antiretroviral activity.

In contrast to our results for HIV-1, all of the HIV-2 and SIV isolates tested in our study were highly resistant to GSK232. Thus, despite the improved inhibitory profile of GSK232 with respect to polymorphic HIV-1 Gag variants (i.e, in comparison to bevirimat) [51], the antiretroviral activity of GSK232 does not appear to extend to HIV-2 and related SIVs. Furthermore, we show that three amino acid changes in the CA/SP1 site of HIV-2_ROD9_ are sufficient to convert the virus to a form that is susceptible to GSK232, with an IC_50_ that is comparable to the values observed for HIV-1. This result is consistent with the aforementioned study of bevirimat resistance in SIV_mac239_ [23]. Our findings for the CA/SP1 mutant of HIV-2_ROD9_ are also concordant with other studies showing that specific amino acid changes in CA/SP1 of HIV-1 can confer resistance to bevirimat [15, 16, 19–21, 43, 44, 49] and to the bevirimat analogs GSK254 and GSK795 [45].

The biochemical basis of intrinsic GSK232 resistance in HIV-2 and SIV is unknown, and any speculation in this regard is complicated by the paucity of data for retroviruses other than HIV-1. However, if we assume that HIV-2 and SIV operate via a mechanism similar to the one proposed for HIV-1, then the simplest model is that GSK232 is unable to bind to the HIV-2 and SIV CA-SP1 proteins. As a result, the formation of a complex in which GSK232 locks CA-SP1 in the stable 6-HB structure does not occur, and HIV-2/SIV maturation proceeds unabated. This model is supported by the finding that SIV_mac239_ particles (specifically, immature, protease-deficient virions) exhibit low levels of bevirimat incorporation when treated with the inhibitor; introduction of the CA/SP1 M3 mutations (equivalent to HIV-2_ROD9_ M3 in our study; Fig 3A) increases bevirimat incorporation by approximately 30-fold [17]. It is also possible that, in the hexameric Gag lattice, the equilibrium state of HIV-2/SIV CA-SP1 is inherently shifted towards the random coil conformation. In this scenario, GSK232 binding occurs in HIV-2 and SIV, but is insufficient to drive the equilibrium towards 6-HB formation. A third possibility is that intrinsic resistance to maturation inhibitors in HIV-2 and SIV involves both diminished drug binding and an inherent favoring of the random-coil form of CA-SP1. Additional studies are needed to address these potential mechanisms.

### Conclusion

Taken together, our data indicate that HIV-2 and related SIVs are intrinsically resistant to the maturation inhibitor GSK232. Our findings also suggest that GSK232 is not a suitable candidate for antiretroviral therapy of HIV-2 infection. There are few studies of HIV-2 maturation in the published literature, and structures of the mature and immature HIV-2 CA lattices are not yet available. Further studies of HIV-2 maturation might lead to compounds with a broader spectrum of antiretroviral coverage, including those with activity against HIV-2.

## Supporting information

S1 Fig Cytotoxicity of GSK232 in CEMss, MAGIC-5A, and 293T/17 cell cultures. Cytotoxicity of GSK232 in MAGIC-5A, 293T/17, and CEMss cells. ATP levels in cell lysates were quantified using the CellTiter-Glo^®^ Luminescent Cell Viability Assay (Promega Corp, Madison, WI) as described in the Materials & methods. Luminescence was quantified using a Victor^3^ Multi-Label plate reader (PerkinElmer Inc., Akron, OH). Cell viability (% of no-drug control) was calculated as the magnitude of the luminescence signal in each culture well relative to the average signal from two cultures that received solvent only. Each datum point is the mean of two values from two assay wells. Error bars indicate ±1 SD and when not visible are smaller than the symbols.
(PDF)

S2 Fig Additional examples of dose-response plots from spreading infections of GSK232-treated CEMss cells. Data points indicate the amount of infectious virus produced in GSK232-treated CEMss cells relative to the amount produced in cultures that received solvent only (no-drug controls). Each point is the mean of four cultures that were maintained in parallel. Error bars indicate ±1 SD and, when not visible, are smaller than the symbols. IC_50_ values were calculated for the HIV-1 isolates using a four-parameter regression model in GraphPad Prism 6.0 as described in the Materials & methods. R^2^ values for the regressions are also shown as calculated in Prism.
(PDF)

S3 Fig Examples of dose-response plots showing the activity of GSK232 against HIV-1 in the single-cycle assay. Data points indicate the amount of infectious virus produced in GSK232-treated 293T/17 cells relative to the amount produced in cultures that received solvent only (no-drug controls). Each point is the mean of four cultures that were maintained in parallel. Error bars indicate ±1 SD and, when not visible, are smaller than the symbols. IC_50_ values were calculated using a four-parameter regression model in GraphPad Prism 6.0 as described in the Materials and Methods. R^2^ values for the regressions are also shown as calculated in Prism.
(PDF)

S4 Fig Examples of dose-response plots showing the activity of GSK232 against HIV-2 and SIVmac in the single-cycle assay. Data points indicate the amount of infectious virus produced in GSK232-treated 293T/17 cells relative to the amount produced in cultures that received solvent only (no-drug controls). Each point is the mean of four cultures that were maintained in parallel. Error bars indicate ±1 SD and, when not visible, are smaller than the symbols.
(PDF)
