# Supplementary figures and images for: Intrinsic resistance of HIV-2 and SIV to the maturation inhibitor GSK2838232
Source: PLoS One. 2023 Jan 18;18(1):e0280568. doi: 10.1371/journal.pone.0280568 (PMC9847912; doi:10.1371/journal.pone.0280568)

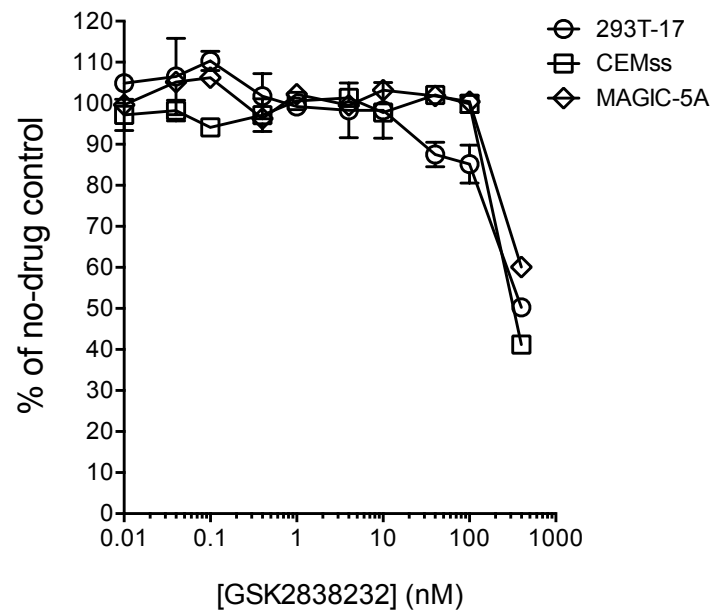

**S1 Fig. Cytotoxicity of GSK232 in MAGIC-5A, 293T/17, and CEMss cells.**

Supplement: S1 Fig — Cytotoxicity of GSK232 in MAGIC-5A, 293T/17, and CEMss cells. ATP levels in cell lysates were quantified using the CellTiter-Glo® Luminescent Cell Viability Assay (Promega Corp, Madison, WI) as described in the Materials & methods. Luminescence was quantified using a Victor3 Multi-Label plate reader (PerkinElmer Inc., Akron, OH). Cell viability (% of no-drug control) was calculated as the magnitude of the luminescence signal in each culture well relative to the average signal from two cultures that received solvent only. Each datum point is the mean of two values from two assay wells. Error bars indicate ±1 SD and when not visible are smaller than the symbols. (PDF) [file pone.0280568.s001.pdf]

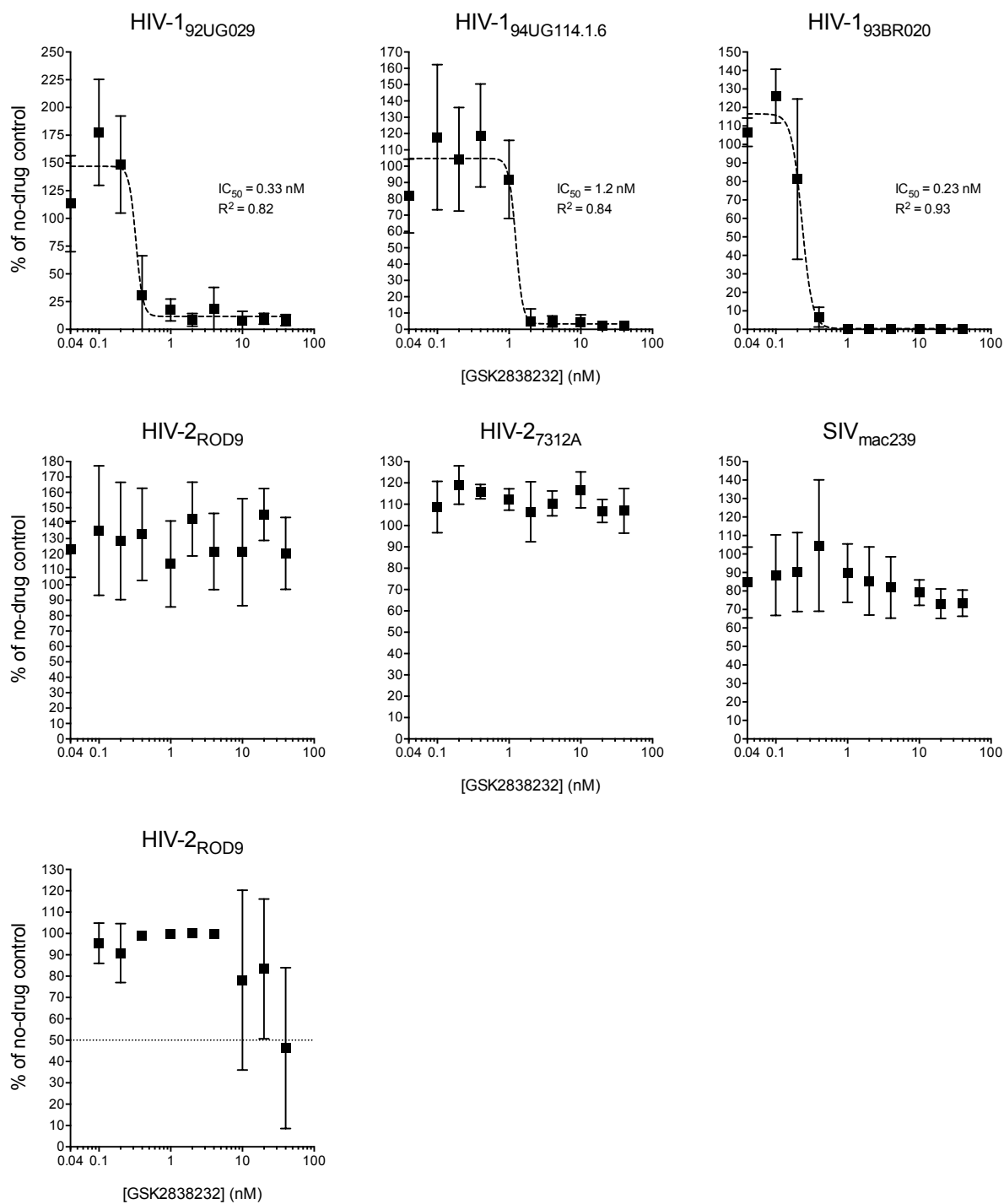

**S2 Fig. Additional examples of dose-response plots from spreading infection assays with GSK232.**

Supplement: S2 Fig — Data points indicate the amount of infectious virus produced in GSK232-treated CEMss cells relative to the amount produced in cultures that received solvent only (no-drug controls). Each point is the mean of four cultures that were maintained in parallel. Error bars indicate ±1 SD and, when not visible, are smaller than the symbols. IC50 values were calculated for the HIV-1 isolates using a four-parameter regression model in GraphPad Prism 6.0 as described in the Materials & methods. R2 values for the regressions are also shown as calculated in Prism. (PDF) [file pone.0280568.s002.pdf]
