# Supplementary material for: Intrinsic resistance of HIV-2 and SIV to the maturation inhibitor GSK2838232
Source: PLoS One. 2023 Jan 18;18(1):e0280568. doi: 10.1371/journal.pone.0280568 (PMC9847912; doi:10.1371/journal.pone.0280568)
Supplement: S4 Fig — Data points indicate the amount of infectious virus produced in GSK232-treated 293T/17 cells relative to the amount produced in cultures that received solvent only (no-drug controls). Each point is the mean of four cultures that were maintained in parallel. Error bars indicate ±1 SD and, when not visible, are smaller than the symbols. (PDF) [file pone.0280568.s004.pdf]

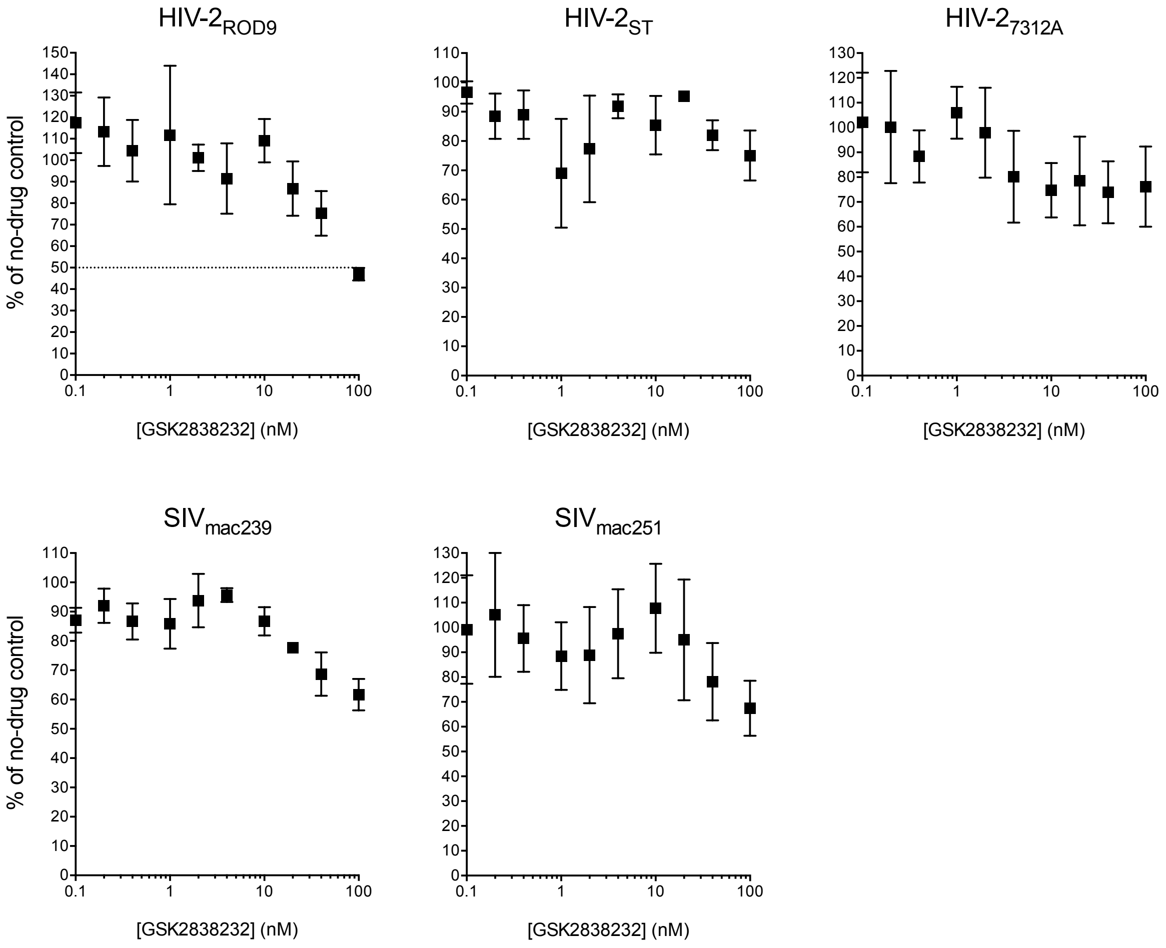

S4 Fig. Examples of dose-response plots showing the activity of GSK232 against HIV-2 and SIV_{mac} in the single-cycle assay.
